# Tumor and immune cell distribution in the tumor core and outer part of glioblastoma, IDH wildtype

**DOI:** 10.1007/s11060-025-05232-5

**Published:** 2025-09-25

**Authors:** Vilde Pedersen, Arnon Møldrup Knudsen, Signe Regner Michaelsen, Rikke Hedegaard Dahlrot, Bjarne Winther Kristensen

**Affiliations:** 1 Department of Pathology, The Bartholin Institute, Rigshospitalet, Copenhagen University Hospital, Copenhagen, Denmark; 2 Department of Clinical Medicine and Biotech Research and Innovation Center (BRIC), University of Copenhagen, Copenhagen, Denmark; 3 DCCC Brain Tumor Center, Rigshospitalet, Copenhagen University Hospital, Copenhagen, Denmark; 4 Department of Pathology, Odense University Hospital, Odense, Denmark; 5 Department of Clinical Research, University of Southern Denmark, Odense, Denmark; 6 Department of Oncology, Odense University Hospital, Odense, Denmark

**Keywords:** Glioblastoma, Periphery, T cells, Tumor-associated microglia and macrophages, TAM, Microenvironment

## Abstract

**Purpose:**

Glioblastoma, IDH-wildtype is the most frequent and malignant primary brain tumor in adults. Tumor cells infiltrate the brain parenchyma, preventing complete resection and causing progression. Immune therapies have limited effect, but little is known about the frequency and type of immune cells in the outer part of glioblastoma, IDH-wildtype, where tumor cells start to infiltrate the brain (transition zone) and diffusely infiltrate the brain parenchyma (tumor periphery). We aimed to quantify the type and distribution of immune cells in glioblastomas, IDH-wildtype covering these areas.

**Methods:**

We established a cohort of 54 glioblastomas, IDH-wildtype containing tissue from the tumor core, transition zone, and periphery. Patients were included if most tumor cells were positive in immunohistochemical staining for P53. Tissue sections were subject to multiplex immunohistochemistry and stained with P53 (tumor), FOXP3 (regulatory T cells), CD8 (cytotoxic T cells), and IBA1 (microglia/macrophages). A software-based classifier was trained to count the cells.

**Results:**

The densities of CD8+, FOXP3+, and IBA1+ cells were significantly higher in the core than in the periphery and in the transition zone than in the periphery. However, the CD8+, FOXP3+, and IBA1+ cell/tumor cell ratio increased from the core to the transition zone, and the CD8+ and IBA1+ cell/tumor cell ratio increased again to the periphery. The core had the highest FOXP3+/CD8+ ratio, as well as the highest fraction of tumor cells with IBA1+ cells, CD8+ cells, and FOXP3+ cells in proximity.

**Conclusion:**

This study highlights spatial differences in the immune microenvironment with potential implications for future immune-therapeutic strategies.

**Graphical abstract:**

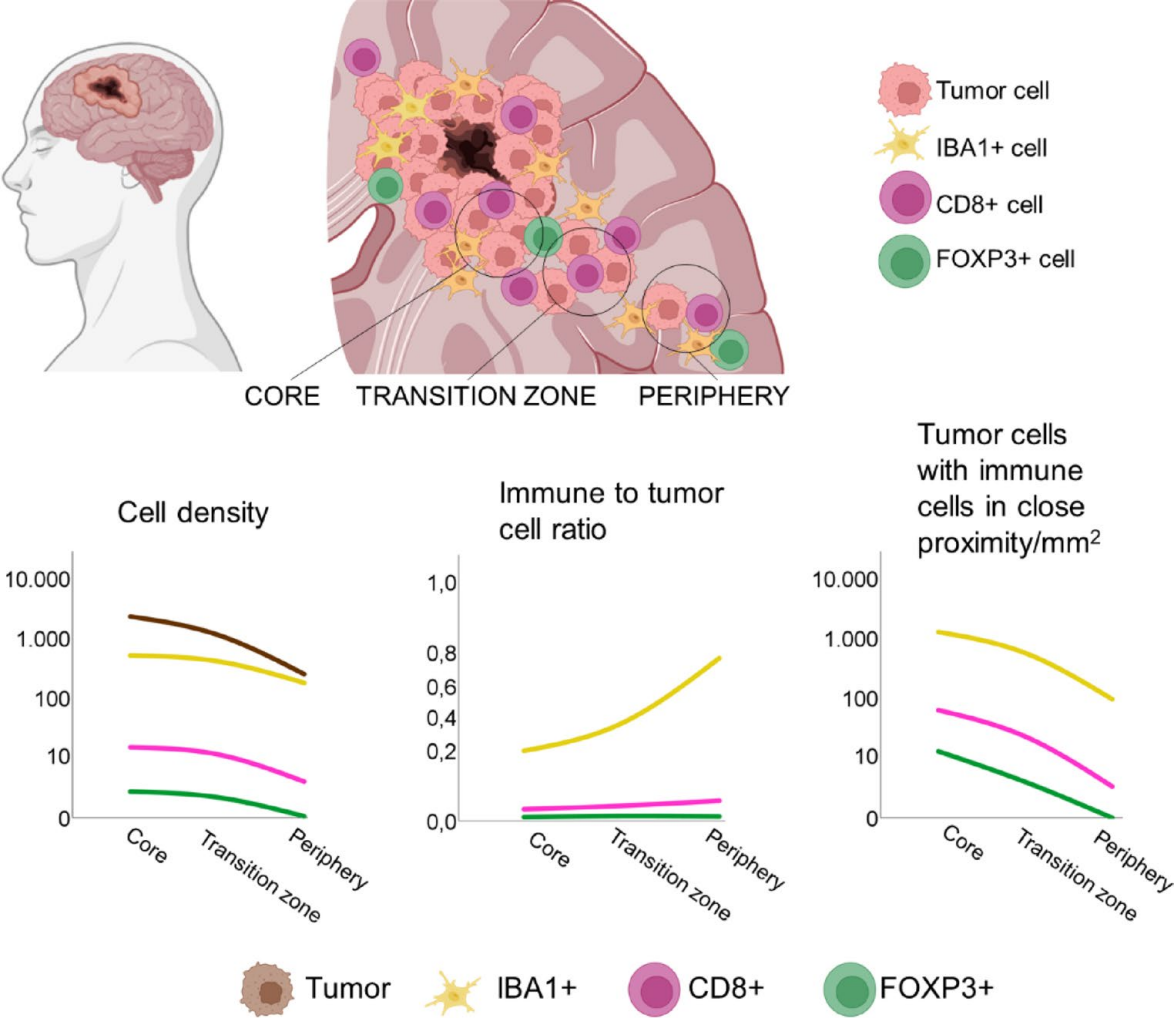

**Supplementary Information:**

The online version contains supplementary material available at 10.1007/s11060-025-05232-5.

## Introduction

Immunotherapies have revolutionized therapy for several different cancer types [1–3], but glioblastoma, the most common and aggressive brain tumor in adults, has shown a minimal response [4, 5]. Glioblastoma cells rapidly and diffusely infiltrate the brain parenchyma, and the infiltrating tumor cells can neither be removed surgically nor be eliminated by radiation and chemotherapy [6–8]. The infiltrating tumor cells in glioblastoma are found in the tumor periphery – the leading edge of the tumor – and the transition zone, located between the tumor core and the periphery. Tumor cells in these regions lead to progression [9], but there is a gap in knowledge about how tumor and immune cells are distributed in these areas [10, 11].

In glioblastomas, the tumor core is densely populated with tumor-associated microglia and macrophages (TAMs), comprising up to 30% of the total cells [12]. T cells are also present in glioblastomas, and despite being largely outnumbered by TAMs, their presence still holds clinical significance. Studies show an association between an increased amount of CD8+ cytotoxic T cells in glioblastoma and improved survival [13, 14]. In contrast, FOXP3+ regulatory T cells, known for their immunosuppressive effects, have been associated with reduced overall survival in glioblastoma [15, 16]. However, Guo et al. [15] reported that 80% of the tumors in the FOXP3-low group are astrocytomas WHO grade 3, which may impact overall survival (OS). Furthermore, there is a lack of treatment information. Additionally, most studies focus on the tumor core and also include IDH-mutant astrocytomas as astrocytoma, IDH-mutant WHO grade 4 was classified as glioblastoma until the publication of the 5th edition of the WHO Classification of Central Nervous System Tumors in 2021. As these tumors have a better prognosis than glioblastoma, IDH-wildtype, previous work may be biased. Recent studies, including omics studies [10, 17], have focused on the periphery, but they often involve a limited number of patients. This limitation arises from the difficulty of obtaining tissue from these outer regions, as surgeons prioritize preserving as much healthy brain tissue as possible. In a study from 2018 based on 10 glioblastoma, IDH-wildtype patients, the quantitative immunohistochemical evaluation of immune cell differences between the tumor core and the periphery showed an increased number of CD8+ cytotoxic T cells in the periphery, while the tumor core exhibited a higher number of FOXP3+ regulatory T cells and TAMs [18].

The intricate interplay of tumor cells, immune cells, and resident brain cells, such as neurons, astrocytes, and oligodendrocytes, appears to be of importance for tumor biology and therapeutic efficacy [19–24]. Tumor cells interact with TAMs [12] and cytotoxic and regulatory T cells [16], with experimental data from our group suggesting that interactions between TAMs and tumor cells promote aggressiveness in glioblastoma [25]. One study on glioblastoma, IDH-wildtype using quantitative immunohistochemistry, found a positive correlation between the expression of anti-inflammatory and tumorigenic TAMs (CD68+, TREM2+, and CD163+ cells) and CD8+ cytotoxic T cells, especially at the tumor border, suggesting that there might be area-specific interactions between these cells in glioblastoma [26].

Distance and proximity between the different cell types may also contribute to the aggressiveness of tumors. Previous results showed an association between an increased number of FOXP3+ regulatory T cells within 30 μm of CD8+ cytotoxic T cells and reduced overall survival in oral squamous cell carcinoma [27] but no data are currently available in glioblastoma, IDH-wildtype.

In the present study, the aim was to compare the tumor core, transition zone, and periphery by quantifying the density of tumor cells, CD8+ cytotoxic T cells, FOXP3+ regulatory T cells, and TAMs. We used our already established P53 mutated glioblastoma periphery cohort [28, 29], where tumor cell identification is possible, even in areas with diffuse tumor cell infiltration. We combined this with a chromogenic 4-plex immunohistochemical staining and a software-based deep learning classifier to enable the identification and quantification of tumor cells, cytotoxic T cells, regulatory T cells, and TAMs in the same histological sections. By this approach, we contribute with insights into the distribution of tumor and immune cells in the tumor core, transition zone, and periphery of glioblastoma, IDH-wildtype.

## Materials and methods

### Patient tissue selection

**Fig. 1:**
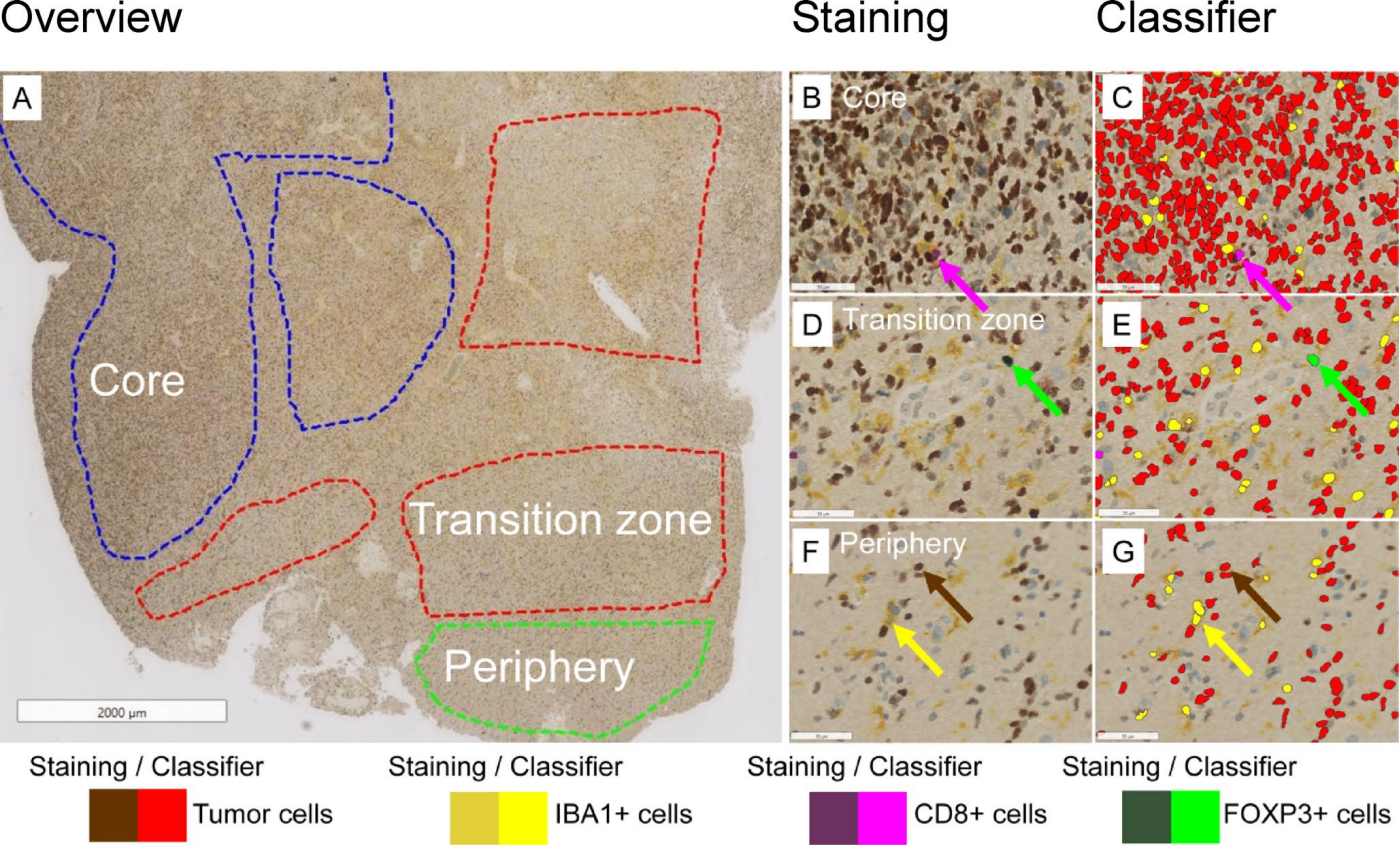
Outlining of regions and quantification of tumor and immune cells. **A** Regions of interest in glioblastoma tissue sections were outlined: tumor core, transition zone, and tumor periphery. **B**, **D**, **F** The sections were stained with four-plex immunohistochemical staining: P53+ tumor cells, IBA1+ microglia/macrophages, CD8+ cytotoxic T cells, and FOXP3+ regulatory T cells. **C**, **E**, **G** Identification of the different cell types with the software-based classifier. The classifier was able to categorize the different cell types into four different classes: P53+ tumor cells, IBA1+ microglia/macrophages, CD8+ cytotoxic T cells, and FOXP3+ regulatory T cells. Scale bar in A = 2000 μm; scale bar B-G = 55 μm

Patient selection and tissue inclusion criteria followed methods previously described by Knudsen et al. [30]. Briefly, archived sections of formaldehyde-fixed, paraffin-embedded tissue from patients diagnosed with primary glioblastoma, IDH-wildtype, at Odense University Hospital, Odense, Denmark, between January 1, 2005, and December 31, 2019, were retrieved from the pathology archive and stained with H&E and P53 immunohistochemistry and reviewed. The inclusion criteria for this cohort, which focuses on changes from the tumor core to the periphery in glioblastomas, were as follows: (1) Confirmed diagnosis of primary glioblastoma, IDH-wildtype, (2) presence of tumor core, transition zone, and/or periphery on the same slide (Fig. 1), (3) P53-positive immunohistochemical reaction in the vast majority of tumor cells. The classification of regions was based on distinct histopathological features observed microscopically. The tumor core was characterized by necrosis, microvascular proliferation, and high tumor cell density, with over 60% of the total cell population showing P53 positivity, indicating tumor cells. The transition zone covered a zone with decreasing tumor cell density and a clear infiltration of brain parenchyma. The periphery was defined by low tumor cell density with single tumor cell infiltration and presence of neurons and/or oligodendrocytes. Region assignment was confirmed by a board-certified neuropathologist (BWK) and further supported by quantified tumor cell density that decreased with distance from the tumor core. A total of 54 patients who met these criteria were included in the study for data analysis. Table 1 provides an overview of the patient characteristics.

**Table 1:** Patient characteristics

Status Dead	
	52
Alive	2
OS (median - range) month	14 (1.7–192)
Status, progression or death	
No	1
Yes	53
PFS (median – range)	7.6 (1.5–153)
Age	
<65	30
>65	24
Sex	
Male	35
Female	19
Performance status	
0–2	49
>2	5
Tumor crossing midline	
Yes	7
None	43
Missing	4
Post-surgical treatment	
Long-course RT+ chemotherapy	40
Palliative	9
None	5
MGMT-status	
Methylated	32
Unmethylated	19
Unknown	3
Surgery	
Biopsy	2
Partial	30
Total	22

### Immunohistochemistry

Tissue sections with a thickness of 3 μm were cut on a microtome and placed on glass slides. They were then subjected to deparaffinization and heat-induced epitope retrieval (HIER) with CC1 buffer for 32 min at 100 °C. After blocking endogenous peroxidase activity, the tissue sections were stained with H&E or incubated with the following antibody: P53 (Clone: DO7, ready-to-use, Ventana Medical Systems) for 32 min at 36 °C. Antibody detection was performed using the Optiview-DAB detection system, and nuclei were counterstained with hematoxylin. This staining was used for screening a larger set of tumors than those included in the cohort.

Tissue sections were prepared as previously described and incubated with FOXP3 antibody (Clone: 236 A/E7, 1:40, ThermoFisher Scientific) for 16 min at 36 °C. Antibody detection was performed with the Discovery-OmniMap-HRP detection system in combination with the DISCOVERY Green HRP Kit and the DISCOVERY HQ-HRP Amp Kit. The tissue sections were then subjected to a new HIER and incubated with P53 antibody (Clone: DO7, Ready-to-use, Ventana Medical Systems) for 8 min at 36 °C. Antibody detection was performed with the Discovery HQ-HRP DAB kit. Following another HIER, the sections were incubated with CD8 antibody (Clone: C8/144B, 1:100, Dako) for 32 min at 36 °C. Antibody detection was performed with Discovery HQ-HRP and the DISCOVERY Purple Kit. Another HIER was performed, and the sections were incubated with IBA1 (1:5000, Wako Pure Chemical Industries) for 16 min at 36 °C. Antibody detection was performed with Discovery HQ-AP and the DISCOVERY Yellow Kit. Finally, nuclei were counterstained with Hematoxylin. All stainings were performed on the Ventana Discovery Ultra platform (Ventana Medical Systems). Two tissue multiblocks served as both negative and positive controls: One containing 27 different normal tissues and 12 different cancers and one containing nine different GBMs. Stained slides were digitalized using the NanoZoomer 2.0HT digital image scanner (Hamamatsu Photonics, Japan).

### Development of a deep learning classifier with visiopharm software module using convolutional neural networks

The digitalized images were imported into Visiopharm software (version 2021.02.5) (Visiopharm, Hoersholm, Denmark). Regions of Interest (ROIs) were delineated based on tumor cell density, which was assessed using P53 immunohistochemical staining: the tumor core, the transition zone, and the tumor periphery (Fig. 1). Areas with bleeding, necrosis, folds in the tissue, and areas with widespread background staining were excluded. ROI selection was approved by an experienced neuropathologist (BWK).

A convolutional neural network was trained at 40X magnification to detect five different cell types: P53+ tumor cells, CD8+ cytotoxic T cells, FOXP3+ regulatory T cells, IBA1+ microglia/macrophages, and unstained cells (Fig. 1). The software was trained on several sections from different patients and zones to account for the intra- and interpatient heterogeneity. The performance of the classifier was assessed manually by thoroughly examining the slides. The classifier was then corrected with several post-processing steps and, in particular, difficult areas, with manual correction.

The number and density of cells were quantified in the different zones. The output variables obtained from the classification were the number and density of P53+ tumor cells, CD8+ cells, FOXP3+ cells, and IBA1+ cells in the different ROIs: tumor core, transition zone, periphery, and normal brain tissue. Additionally, to explore the potential interaction between cells, the cell density of tumor cells with immune cells in close proximity was assessed by quantifying the density of a reference cell type and determining the number of cells within a 30 μm radius of this reference. As labeling cells before quantification is mandatory, we opted to categorize the number of cells in proximity to the reference cell into three groups: 1–4 cells, 5–9 cells, or 10 or more cells.

Batch analyses were performed, and results were exported into an Excel library.

### Statistical analysis

Due to the non-Gaussian distribution of the data, cell density comparisons were made using the Wilcoxon Signed Ranks Test. The univariate relationship between parameters and progression-free survival (PFS) and overall survival (OS) was compared using univariate analyses. For all analyses, the median was used as a pre-specified cut-off. PFS was defined as the time from initial surgery until progression or censoring; OS was defined as the time from initial surgery until death or censoring. Censoring was on September 1, 2024. Statistical analyses were conducted using IBM SPSS Statistics for Windows, version 25 (IBM Corp., Armonk, NY, USA) and STATA15 (StataCorp LP, College Station, TX).

## Results

### Patient characteristics

At the time of censoring, two patients were still alive (3.7%). The median OS was 14 months (range 1.7–192 months), while the median PFS was 7.6 months (range 1.5–153 months). Among the 54 patients included in the study, 30 (56%) were under 65 years of age, and 24 (44%) were 65 or older. The male-to-female ratio was 1.8:1. Post-surgical treatment with long-course radiotherapy and chemotherapy was administered to 40 patients (74%), whereas 9 received palliative care, and 5 received no treatment (Table 1).

### Density of tumor cells

**Fig. 2:**
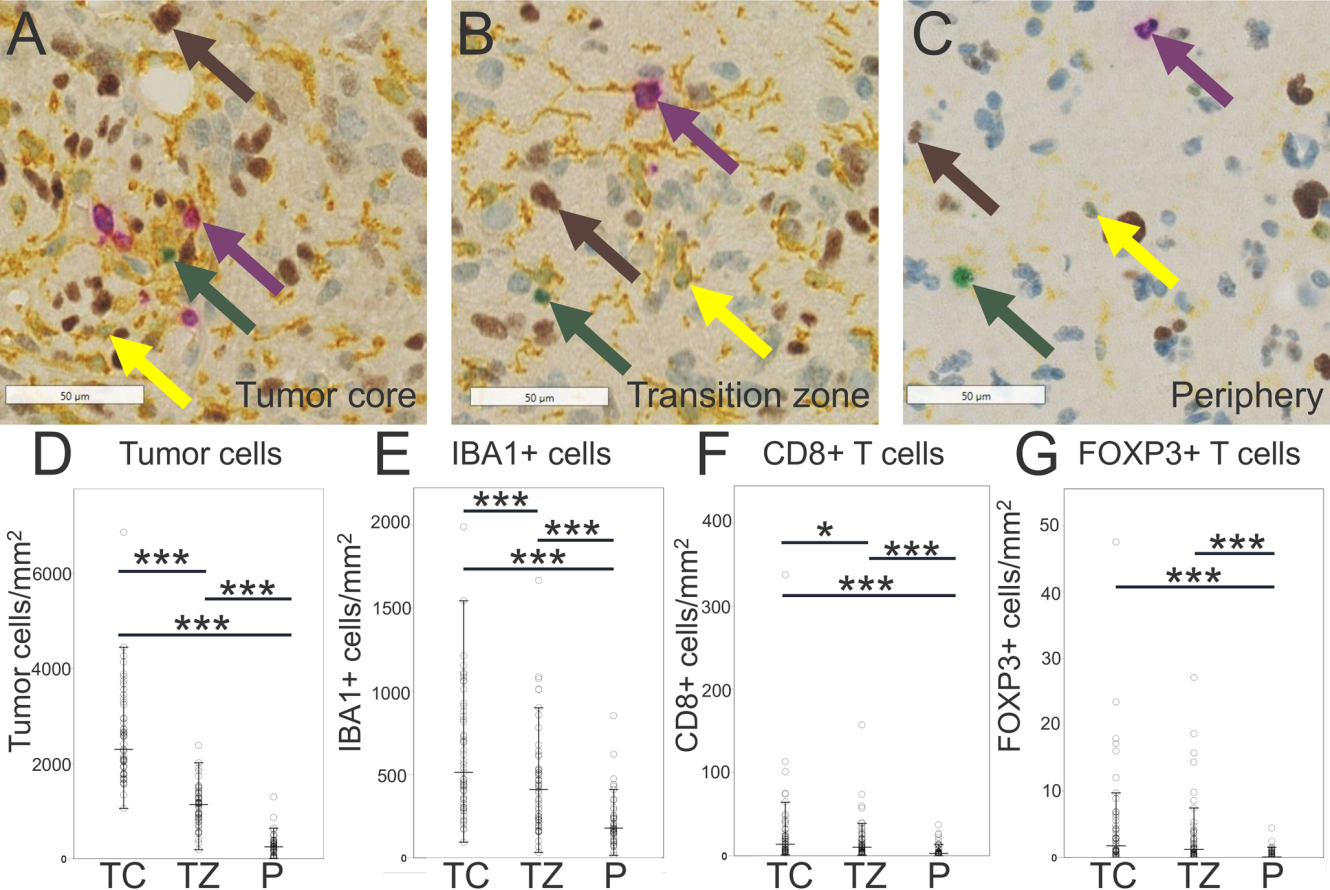
Density of tumor cells and immune cells. **A**–**C**: Representative staining in the tumor core (**A**), transition zone (**B**), and periphery (**C**). **D**–**G**: Expression levels of tumor cell (**D**), IBA1+ microglia-macrophages (**E**), CD8+ T cells (**F**), and FOXP3+ T cells (**G**). The densities of tumor cells, IBA1+ cells, CD8+ cells, and FOXP3+ cells were significantly higher in the core than in the periphery, and in the transition zone than in the periphery. The asterisks indicate the level of significance (*= *P* < 0.05, *** = *P* < 0.001). The arrows indicate tumor cells (brown), IBA1+ cells (yellow), CD8+ cells (purple), and FOXP3+ cells (green). Scale bar = 50 μm. *TC* tumor core, *TZ* transition zone, *P* periphery

**Fig. 3:**
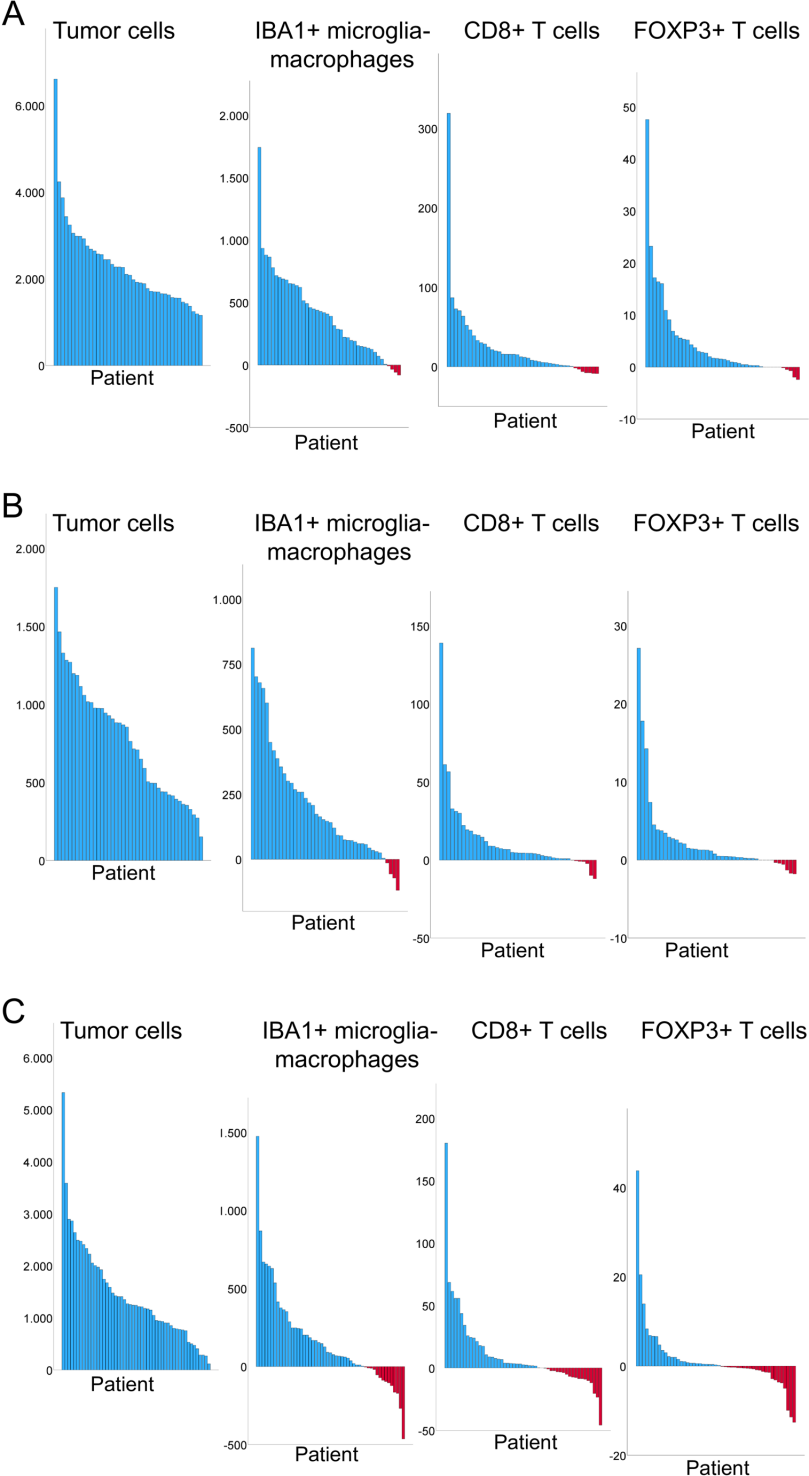
Waterfall plot illustrating the differences in cell densities for individual patients. Each column represents a single patient, with the height of the column indicating the difference in cell densities: **A** density in the tumor core minus the periphery, **B** density in the transition zone minus the periphery, and **C** density in the tumor core minus the transition zone. Blue columns indicate positive values and red columns negative values

The density of tumor cells decreased from the tumor core to the transition zone and again to the periphery (median 2301, 1141, and 250 cells/mm^2^) *P* < 0.001) (Figs. 1 and 2A–D). High interpatient tumor cell heterogeneity was observed, with a higher tumor cell density in the tumor core than in the tumor periphery, a higher tumor cell density in the transition zone compared to the tumor periphery, and a higher tumor cell density in the tumor core than in the transition zone (Fig. 3). These quantitative data showing a decrease in tumor cell density from core to periphery supported the histological selection and outlining of the three distinct tumor regions.

### Density of immune cells

The density of IBA1+ cells was highest in the core, with median values of 512 cells/mm² in the core, 419 cells/mm² in the transition zone, and 179 cells/mm² in the periphery (*P* < 0.001) (Fig. 2A–C and E). Similarly, the density of CD8+ cells was highest in the core, (median 14, 10, and 3 cell/mm²) (*P* < 0.001) (Fig. 2A–C and F). Likewise, FOXP3+ cells exhibited the highest density in the tumor core (median 1.7, 1.2, and 0.1 cells/mm^2^) (*P* < 0.001) (Fig. 2A–C and G).

High interpatient immune cell heterogeneity was observed (Fig. 3). While the majority of patients exhibit the highest densities of immune cells in the tumor core, some patients demonstrate a higher density in the tumor periphery compared to the tumor core (red columns, Fig. 3A), while others show a higher density in the periphery compared to the transition zone (red columns, Fig. 3B), and others show a higher density in the transition zone compared to the tumor core (Fig. 3C).

### Immune cell-to-tumor cell ratios

**Fig. 4:**
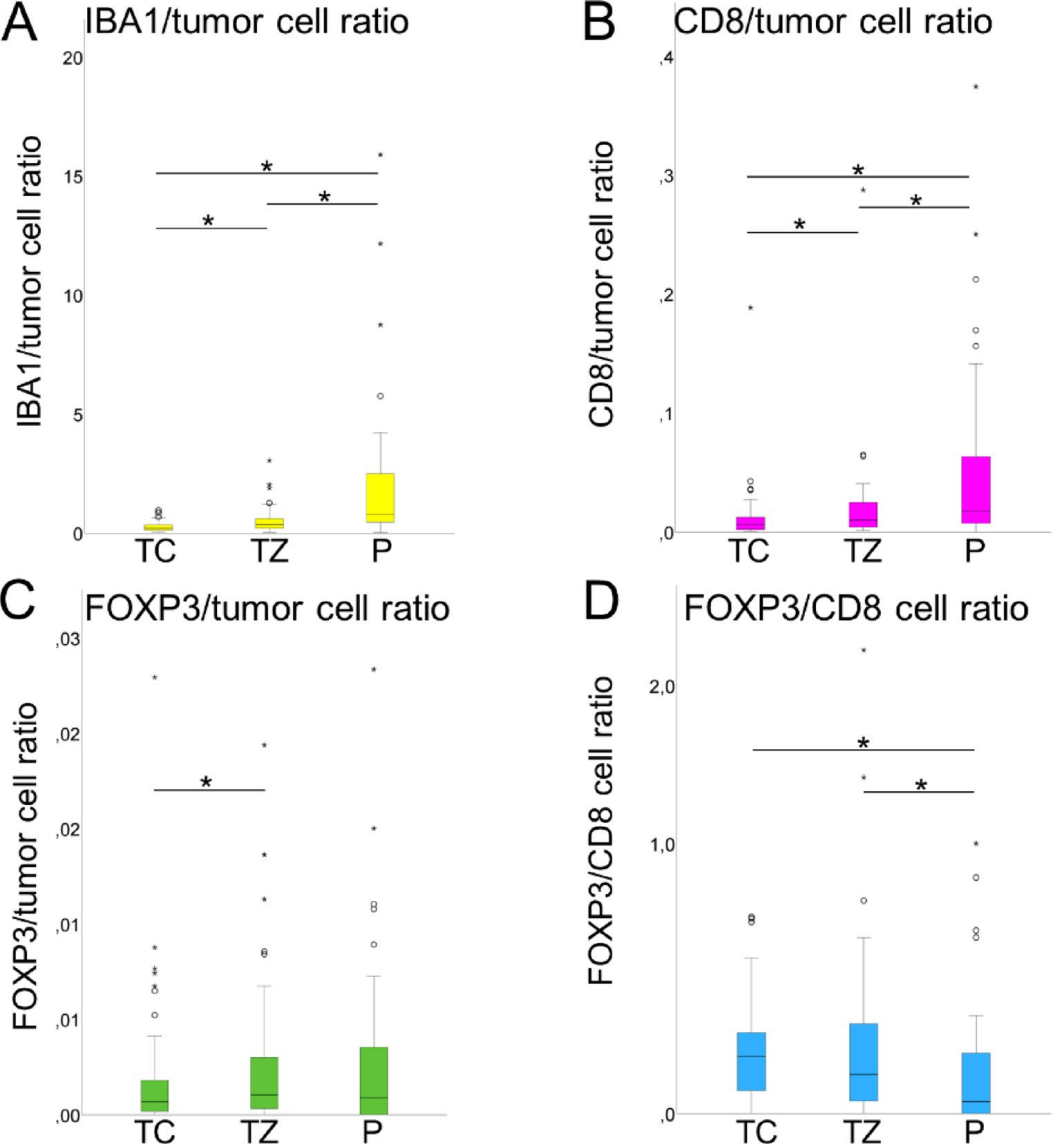
Ratios of immune cell density to tumor cell density in different tumor regions. **A**–**C** The IBA1+/tumor cell ratio (**A**), CD8+/tumor cell ratio (**B**), and FOXP3+/tumor cell ratio (**C**) were higher in the transition zone compared to the tumor core. The IBA1+/tumor cell ratio and CD8+/tumor cell ratio were also significantly higher in the tumor periphery compared to the tumor core. **D** The FOXP3+/CD8+ ratio was significantly higher in the tumor core than in the tumor periphery and significantly higher in the transition zone than in the tumor periphery. Error bars: 95% CI. * = *P* < 0.05. *TC*  tumor core, *TZ* transition zone, *P* periphery

The IBA1+ cell/tumor cell ratio increased from the core to the transition zone and again to the periphery (median values 0.2 in the core, 0.4 in the transition zone and 0.8 in the periphery, *P* < 0.05) (Fig. 4A).

The CD8+ cell/tumor cell ratio was also highest in the periphery (median values 0.006 in the core, 0.010 in the transition zone and 0.018 in the periphery, *P* < 0.05) (Fig. 4B).

The FOXP3+ cell/tumor cell ratio was significantly higher in the transition zone than in the core (0.001 vs. 0.0007, *P* = 0.013) but decreased from the transition zone to the periphery, although not statistically significant (0.001 vs. 0.0009, *P* = 0.669) (Fig. 4C).

### FOXP3+/CD8+ cell ratio

The FOXP3+/CD8+ ratio, which is a measure of the degree of immunosuppression [31–33], was significantly higher in the tumor core than in the tumor periphery (0.16 vs. 0.03, *P* < 0.05) and significantly higher in the transition zone than in the tumor periphery (0.11 vs. 0.3, *P* < 0.05) (Fig. 4D). There was no statistical difference between the tumor core and the transition zone.

### The fraction of tumor cells with immune cells in close proximity to tumor cells

**Fig. 5:**
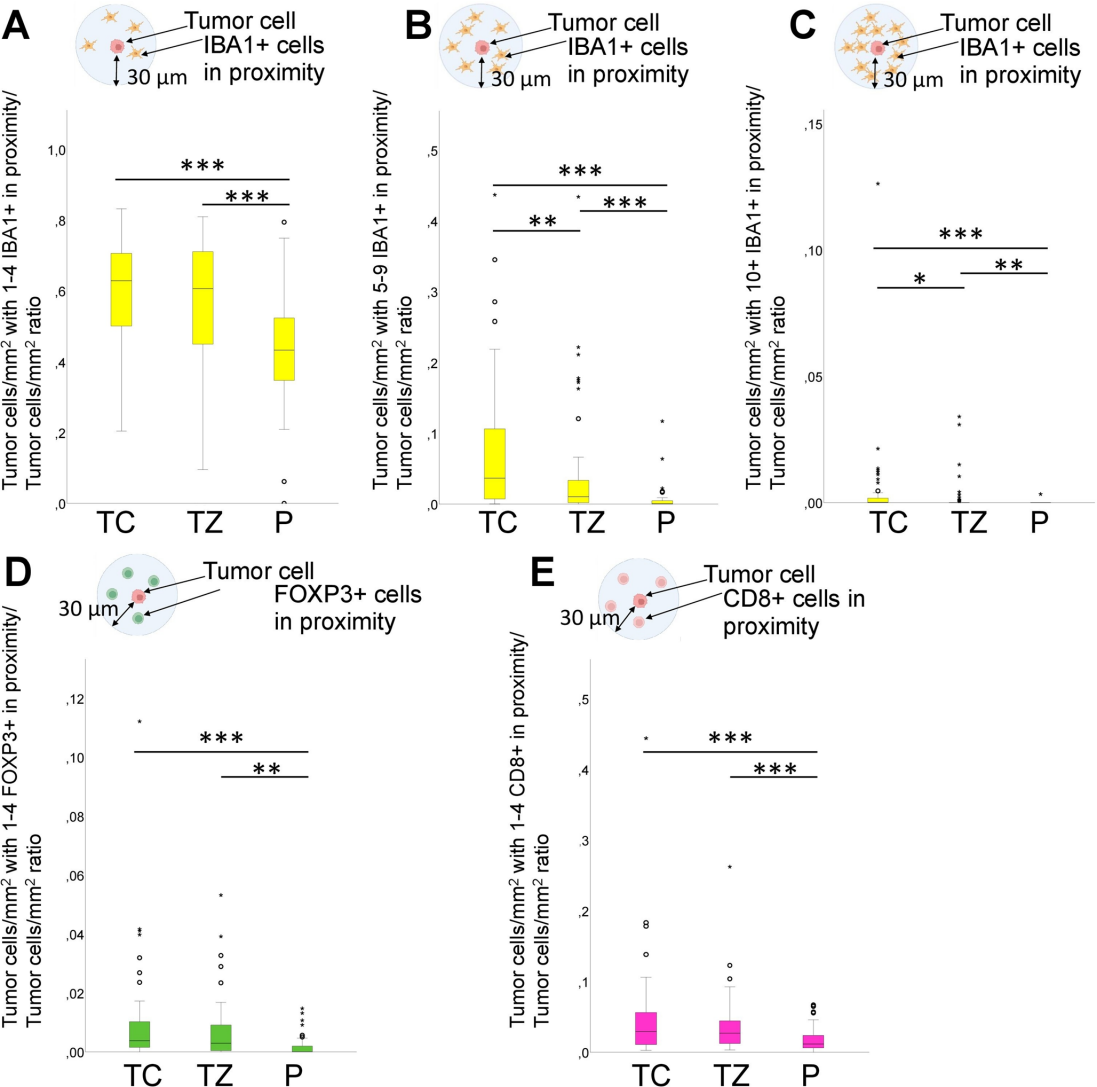
Fraction of the tumor cells with immune cells in close proximity to tumor cells. **A**–**C** The fractions of tumor cells with 1–4 IBA1+ cells, 5–9 IBA1+ cells, and over 10 IBA1+ cells to the total tumor cells were highest in the tumor core. The fraction of tumor cells with 1–4 IBA1+ in proximity to total tumor cells showed no statistically significant difference between the tumor core and the transition zone. **D** The fraction of tumor cells with FOXP3+ cells to the tumor cells was highest in the tumor core. However, the tumor core and the transition zone did not differ significantly. **E** The fraction of tumor cells with CD8+ cells to the tumor cells was highest in the tumor core. No statistically significant difference was observed between tumor core and transition zone. Error bars: 95% CI. * = *P* < 0.05, ** = *P* < 0.01, *** = *P* < 0.001. *TC* tumor core, *TZ* transition zone, *P* periphery

The fraction of tumor cells accompanied by 1–4 IBA1+ cells in close proximity (within 30 μm) to tumor cells revealed that the tumor core exhibited the highest fraction (0.63 in the core, 0.61 in the transition zone, and 0.43 in the periphery). However, there was no statistically significant difference between the tumor core and the transition zone (*P* = 0.09) (Fig. 5A). When examining the fraction of tumor cells accompanied by 5–9 IBA1+ cells in close proximity to tumor cells, we obtained similar findings (0.04 in the tumor core, 0.01 in the transition zone, and 0.0004 in the tumor periphery, *P* < 0.01) (Fig. 5B). For the fraction of tumor cells accompanied by 10 or more IBA1+ cells in close proximity to tumor cells, the median fraction was highest in the tumor core. However, the fractions were very small (0.0002 in the tumor core, 0 in the transition zone, and 0 in the periphery) (Fig. 5C) (*P* < 0.05).

The fraction of tumor cells with 1–4 FOXP3+ cells in close proximity to the tumor cells was highest in the tumor core (0.004 in the core, 0.003 in the transition zone, and 0 in the periphery) (Fig. 5D). However, there was no statistical difference between the tumor core and the transition zone.

For the fraction of tumor cells with 1–4 CD8+ cells in close proximity to the tumor cells, the highest fraction was found in the tumor core (0.029 in the tumor core, 0.027 in the transition zone, and 0.011 in the tumor periphery, but no statistical difference between the tumor core and the tumor periphery). (Fig. 5E).

In the analysis, it was observed that the statistical results remained largely similar, with only minor variations, when evaluating the density of tumor cells with immune cells in close proximity, without calculating the fraction of the total tumor cell density (Supp Fig. 1). We could hereby confirm that the observed patterns were independent of the number of tumor cells.

### Immune cell density, survival, and progression-free survival

The density of the immune cells was not associated with PFS or OS (Supplementary Table S1 and S2). Interestingly, the high density of tumor cells in the core was associated with a lower OS, as indicated by a hazard ratio (HR) of 0.46 (95% CI: 0.26–0.80, *P* = 0.006) (Supplementary Table S1). When examining the density of tumor cells with immune cells in close proximity, a high density of tumor cells with 1–4 IBA1+ cells in close proximity in the transition zone exhibited a near-significant association with improved overall survival (HR = 0.56, *P* = 0.05). No other proximity variables were associated with overall survival or progression-free survival.

## Discussion

In this study, we quantified for the first time the presence, distribution, and proximity of tumor cells and immune cells in the tumor core, transition zone, and periphery of glioblastoma, IDH-wildtype. We used a cohort of over 50 patients with reliable tumor cell identification, utilizing the expression of P53 in tumor cells. Our study is based on multiplex immunohistochemistry followed by quantification by an AI-based software classifier. We outlined the tumor core, transition zone, and periphery, and our data supported that there was a gradual decrease in the density of tumor cells from the core over the transition zone to the periphery.

Our analysis of the distribution of immune cells showed that the densities of all three types of immune cells were highest in the tumor core and lowest in the tumor periphery. Tamura et al. [18] found the number of CD8+ T cells to be highest in the tumor periphery through quantification across three high-power fields, yielding a limited number of cells. This limitation is important, given that T cells constitute only 1–2% of the total cell population in glioblastoma, IDH-wildtype, making this finding potentially less representative [34–36]. In a single-cell RNA sequencing study on samples from four patients, collected from the tumor core and the tumor periphery [10], the number of T cells in the core and periphery was not compared, probably because only a few cells from the tumor periphery were sequenced, illustrating that cell distributions of less frequent cells are challenging to investigate. In contrast, our study was based on 54 patients, where we quantified all cells in the whole tumor core and the periphery.

By quantifying cell-to-cell ratios between immune cells and tumor cells, we found that the CD8+ and IBA1+ cell-to-tumor cell ratios increased from core to transition zone and periphery. Since this ratio is difficult to interpret we quantified the fraction of tumor cells with immune cells in close proximity. The core displayed the highest fraction of tumor cells with IBA1+ cells in close proximity to tumor cells and the highest fraction of tumor cells with FOXP3+ cells in close proximity to tumor cells. This suggests, together with the highest FOXP3+/CD8+ cell ratio, that the tumor core is an area with a high level of immune suppression. FOXP3+/CD8+ ratio has previously been found to be elevated in lymph nodes from patients with metastatic lung adenocarcinoma compared to patients without metastases [37], and a high ratio at the invasive margin has been associated with poorer survival in gastroesophageal adenocarcinomas [31]. Furthermore, in non-small cell lung cancer, a low FOXP3+/CD8+ ratio has been identified as an independent predictor of better response to PD-1 blockade [38]. This suggests that a high FOXP3+/CD8+ ratio is linked to immunosuppressive mechanisms, where FOXP3+ cells inhibit CD8+ cells from initiating an effective immune response. It is uncertain to which degree this may play a role in glioblastoma since the fraction of tumor cells with FOXP3+ (0.004) and CD8+ cells (0.029) in close proximity is very low. Further insights may be gained through studies using larger patient tissue cohorts. Additionally, functional studies using animal models could provide valuable information on immune cell dynamics and their relative ratios.

We have earlier demonstrated that CD204+ TAMs were associated with poor survival in patients treated with temozolomide and irradiation [39] and in a recent study we found that microglia cells protect patient-derived tumor cells growing as spheroids from temozolomide through an interferon-based signaling mechanism [25]. Our findings in the present study reveal a high fraction of tumor cells in close proximity to IBA1+ cells—63% of tumor cells in the core had 1–4 IBA1+ cells nearby. This suggests that microglia-macrophages may more efficiently protect tumor cells from temozolomide in the core compared to the periphery, where 43% of tumor cells had 1–4 IBA1+ cells in close proximity. Nevertheless, there is still a considerable presence of preferentially microglia in this region.

The intact blood-brain barrier - at least in the periphery - might additionally contribute to prevent temozolomide from being effective in this region.

In our well-characterized cohort, no associations were found between the densities of various immune cells and OS or PFS. Numerous studies have investigated the association of the CD8+ T-cell population and survival in glioblastomas. By evaluating immunohistochemistry, Yang et al. found that long survival was associated with an increased CD8+ T-cell infiltration [13]. However, this study was performed when astrocytomas, IDH-mutant WHO grade 4, were diagnosed as glioblastomas, and IDH status was not investigated in the patients. Another study also showed that a high number of cytotoxic T cells correlated with a longer survival in primary glioblastomas [14], although the authors did not specify the IDH-status, with its inherent limitations. Increasing our cohort size could help clarify this and also provide insights into the role of cytotoxic T cells in the outer regions of glioblastoma.

A limitation of the present study is that we focus on P53+ glioblastomas since previous studies have demonstrated that *TP53* gene mutations are absent in glioblastomas of the classical subtype [40]. However, establishing a cohort with P53+ glioblastoma, IDH-wildtype, made it possible for the first time to accurately quantify the distribution of tumor cells and immune cells in the core, transition zone, and periphery of more than 50 patients.

In the present study, we did not perform stainings for glioma stem cell markers. This would be an important aspect to incorporate in future spatial profiling and immunohistochemical multiplexing studies, since malignant cells residing in infiltrated brain tissue have increased expression of genes related to neurodevelopmental pathways, as found in a recent publication from our group [17]. Further insights into the interaction of stem-like glioma cells with immune cells would also be of critical importance for the immune therapy area.

## Conclusion

The densities of tumor and immune cells decreased from the tumor core to the periphery. The core exhibited the highest level of immunosuppression with the highest fractions of tumor cells in proximity to immune cells (CD8+ cells, FOXP3+ cells, and IBA1+ cells) and the highest FOXP3+/CD8+ ratio. However, the tumor periphery still contained a substantial fraction of tumor cells in close proximity to microglial cells. These findings illustrate spatial differences in the immune microenvironment, which may have implications for future therapeutic strategies.

## Supplementary Information

Below is the link to the electronic supplementary material.

Supplementary Material 1

## Data Availability

The data used and analyzed in this study can be obtained from the corresponding author upon reasonable request. However, raw data cannot be freely shared due to restrictions imposed by informed consent and regulations under Danish and EU law. The material is archived at the University of Southern Denmark and is subject to the same restrictions.
