# Supplementary material for: Tumor and immune cell distribution in the tumor core and outer part of glioblastoma, IDH wildtype
Source: J Neurooncol. 2025 Sep 25;175(3):1187–98. doi: 10.1007/s11060-025-05232-5 (PMC12511171; doi:10.1007/s11060-025-05232-5)
Supplement: Supplementary file 1 — Supplementary Material 1 [file 11060_2025_5232_MOESM1_ESM.docx]

**Supplementary**

**Supplementary figure 1**

**
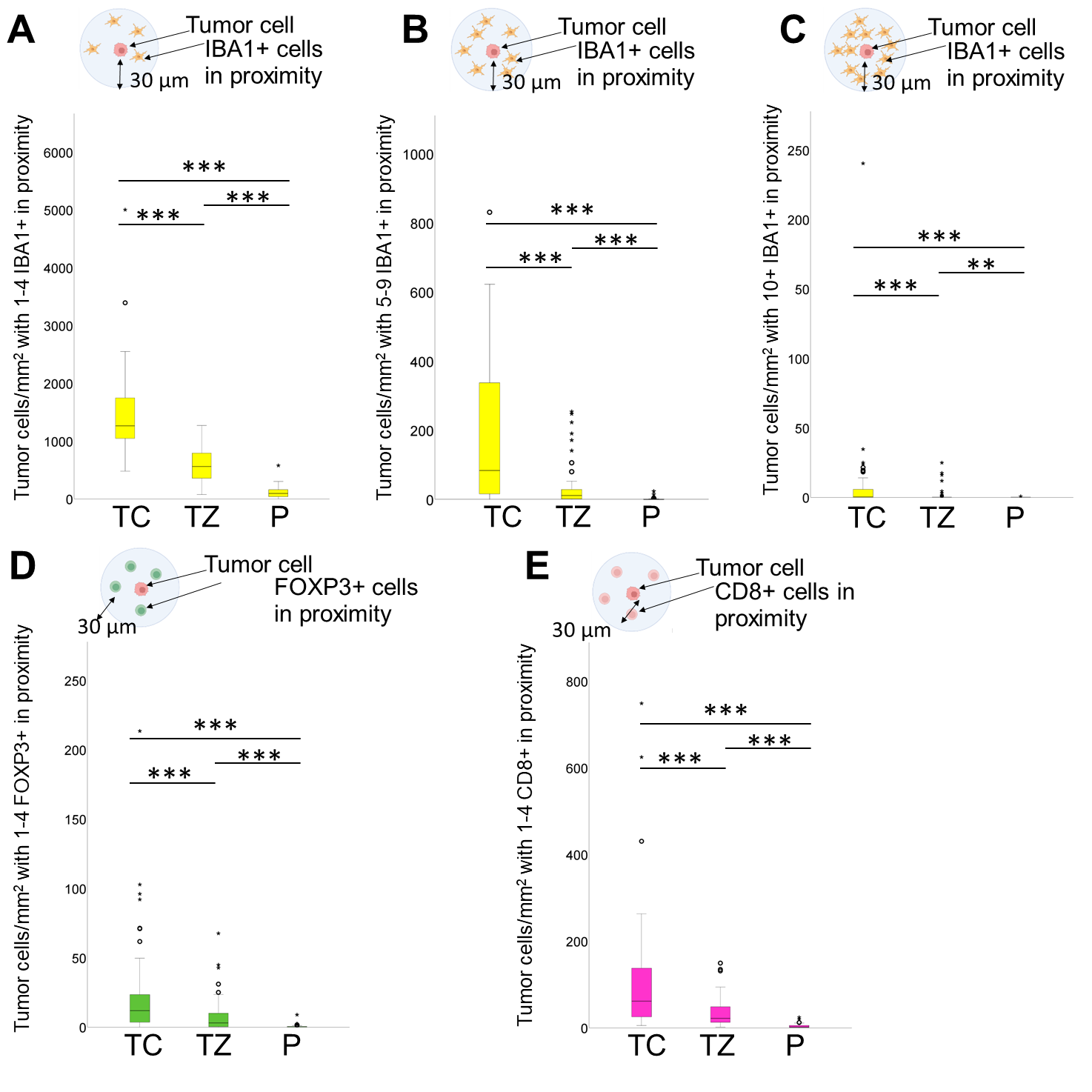
**

**Supp fig. 1:** Tumor cell density with immune cells in close proximity: **(A-C)** The densities of tumor cells with 1-4 IBA1+ cells, 5-9 IBA1+ cells, and 10 or more IBA1+ cells were highest in the tumor core. **(D)** Density of tumor cells with FOXP3+ cells was highest in the tumor core. **(E)** Density of tumor cells with CD8+ cells was highest in the tumor core. Error bars: 95 % CI. * = P <0.05, ** = P <0.01, *** = P < 0.001. TC = tumor core, TZ = transition zone, P = periphery.

**Supplementary figure 2**

**
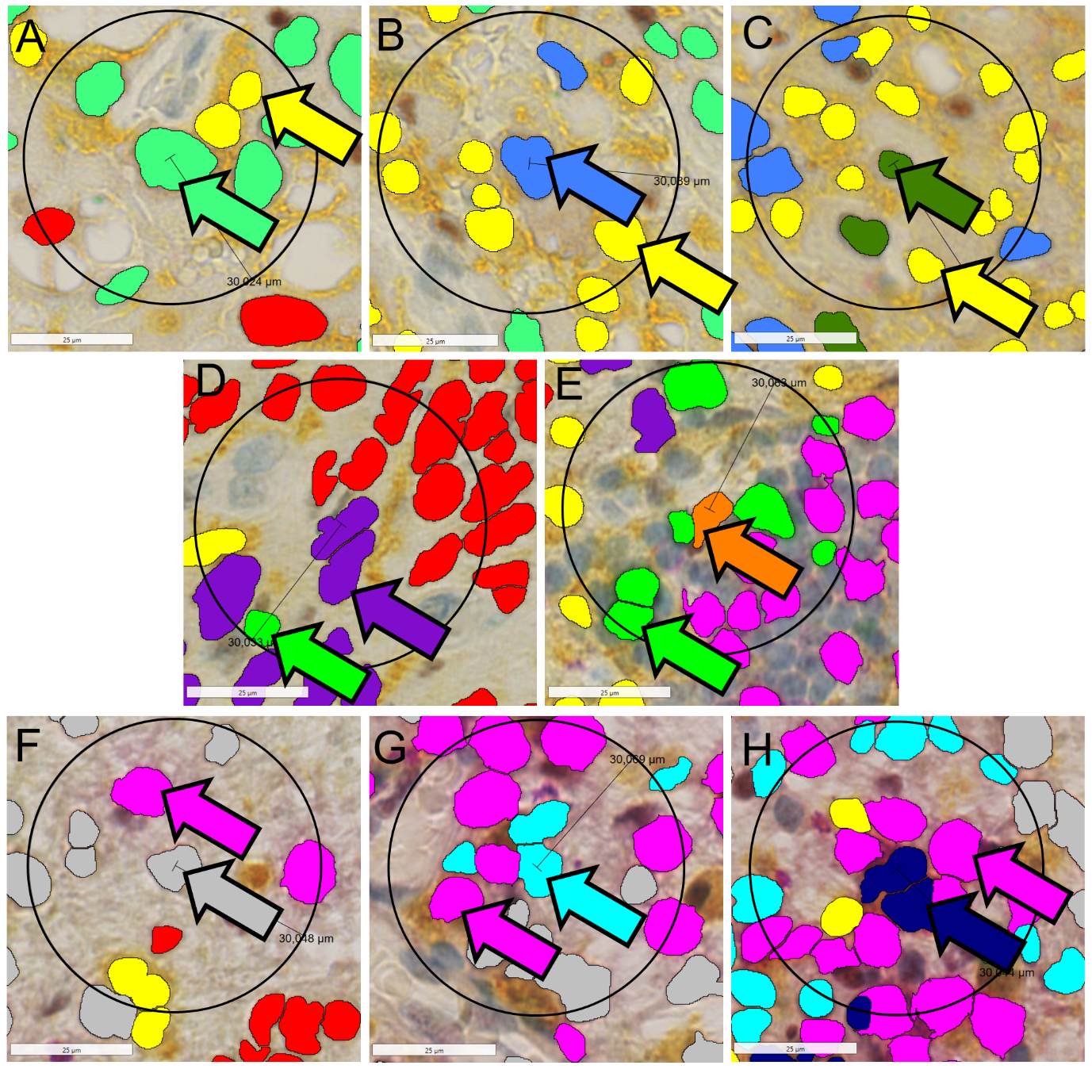
**

**Supp fig. 2: Images illustrating the classifier used in the analysis for Figure 5.**

Images from Visiopharm illustrating the proximity analysis. (**A**) Tumor cells labeled light green (light green arrow), indicating the presence of 1–4 IBA1+ cells (yellow cells – yellow arrow) within 30 µm. (**B**) Tumor cells labeled blue (blue arrow), indicating the presence of 5–9 IBA1+ cells in proximity. (**C**) Tumor cells labeled military green (military green arrow), indicating the presence of more than 10 IBA1+ cells nearby. (**D**) Tumor cells labeled purple (purple arrow), indicating the presence of 1–4 FOXP3+ cells (fluorescent green -fluorescent green arrow) in proximity. (**E**) Tumor cell labeled orange (orange arrow), indicating the presence of 5-9 FOXP3+ cells (fluorescent green arrow) in proximity. (**F**) Tumor cells labeled gray (gray arrow), indicating the presence of 1-4 CD8+ cells (magenta – magenta arrow) in proximity. (**G**) Tumor cells labeled cyan (cyan arrow), indicating the presence of 5-9 CD8+ cells (magenta – magenta arrow) in proximity. (**H**) Tumor cells labeled dark blue (dark blue arrow), indicating the presence of 10 or more CD8+ cells (magenta – magenta arrow) in proximity. Tumor cells that do not have immune cells within 30 µm are red. The black circle surrounding the reference cell has an approximate radius of 30 µm. Scalebar = 25 µm.

**Table S1**

Overall survival dichotomized at the median

| Variable | HR | 95% CI | p-value | Number of patients |
| --- | --- | --- | --- | --- |
| **Cell density** |  |  |  |  |
| Tumor cells/mm^2^ core | 0.46 | 0.26-0.80 | 0.006 | 53 |
| Tumor cells/mm^2^ transition zone | 0.78 | 0.45-1.36 | 0.38 | 52 |
| Tumor cells/mm^2^ periphery | 1.01 | 0.55-1.87 | 0.97 | 43 |
| CD8+ cells/mm^2^ core | 0.85 | 0.49-1.47 | 0.56 | 53 |
| CD8+ cells /mm^2^ transition zone | 0.73 | 0.41-1.27 | 0.26 | 52 |
| CD8+ cells/mm^2^ periphery | 1.19 | 0.63-2.24 | 0.60 | 43 |
| FOXP3+ cells/mm^2^ core | 1.20 | 0.69-2.10 | 0.52 | 53 |
| FOXP3+ cells/mm^2^ transition zone | 0.93 | 0.54-1.63 | 0.81 | 52 |
| FOXP3+ cells/mm^2^ periphery | 0.67 | 0.36-1.26 | 0.21 | 43 |
| IBA1+ cells/mm^2^ core | 1.53 | 0.87-2.69 | 0.14 | 53 |
| IBA1+ cells/mm^2^ transition zone | 0.84 | 0.48-1.46 | 0.53 | 52 |
| IBA1+ cells/mm^2^ periphery | 0.86 | 0.46-1.60 | 0.64 | 43 |
| **Tumor cell proximity** |  |  |  |  |
| Tumor cells/mm^2^ with 1-4 CD8+ cells in proximity, core | 1.10 | 0.63-1.93 | 0.73 | 53 |
| Tumor cells/mm^2^ with 1-4 CD8+ cells in proximity, transition zone | 0.87 | 0.50-1.53 | 0.64 | 50 |
| Tumor cells/mm^2^ with 1-4 CD8+ cells in proximity, periphery | 1.54 | 0.83-2.87 | 0.17 | 43 |
| Tumor cells/mm^2^ with 1-4 FOXP3+ cells in proximity, core | 1.18 | 0.67-2.07 | 0.56 | 53 |
| Tumor cells/mm^2^ with 1-4 FOXP3+ cells in proximity, transition zone | 0.88 | 0.51-1.55 | 0.67 | 52 |
| Tumor cells/mm^2^ with 1-4 FOXP3+ cells in proximity, periphery | 0.93 | 0.54-1.62 | 0.81 | 54 |
| Tumor cells/mm^2^ with 1-4 IBA1+ cells in proximity, core | 1.11 | 0.63-1.95 | 0.72 | 53 |
| Tumor cells/mm^2^ with 1-4 IBA1+ cells in proximity, transition zone | 0.56 | 0.32-1.00 | 0.05 | 52 |
| Tumor cells/mm^2^ with 1-4 IBA1+ cells in proximity, periphery | 1.10 | 0.59-1.03 | 0.78 | 43 |
| Tumor cells/mm^2^ with 5-9 IBA1+ cells in proximity, core | 1.37 | 0.78-2.42 | 0.28 | 53 |
| Tumor cells/mm^2^ with 5-9 IBA1+ cells in proximity, transition zone | 0.67 | 0.38-1-17 | 0.16 | 52 |
| Tumor cells/mm^2^ with 5-9 IBA1+ cells in proximity, periphery | 0.92 | 0.50-1.72 | 0.81 | 43 |
| Tumor cells/mm^2^ with over 10 IBA1+ cells in proximity, core | 1.10 | 0.63-1.90 | 0.74 | 53 |
| Tumor cells/mm^2^ with over 10 IBA1+ cells in proximity, transition zone | 0.63 | 0.34-1.16 | 0.14 | 54 |
| Tumor cells/mm^2^ with over 10 IBA1+ cells in proximity, periphery | All recorded measurements are 0. |  |  |  |

Tumor cells/mm^2^ with over 10 IBA1+ cells in proximity, transition zone: A substantial number of cases have a value of 0. We have categorized them into two groups: 0 and >0. The group with a score of 0 comprises 40 cases, while the group with a higher score includes 13 cases.

Tumor cells/mm^2^ with 1-4 FOXP3+ cells in proximity, periphery: A substantial number of cases have a value of 0. We have categorized them into two groups: 0 and >0. The group with a score of 0 comprises 26 cases, while the group with a higher score includes 28 cases.

**Table S2**

Progression-free survival dichotomized at the median

| Variable | HR | 95% CI | p-value | Number patients |
| --- | --- | --- | --- | --- |
| **Cell density** |  |  |  |  |
| **Tumor cells/mm^2^ core** | **0.60** | **0.35-1.05** | **0.07** | **53** |
| Tumor cells/mm^2^ transition zone | 1.00 | 0.58-1.74 | 0.99 | 52 |
| Tumor cells/mm^2^ periphery | 1.26 | 0.68-2.32 | 0.46 | 43 |
| CD8+ cells/mm^2^ core | 0.67 | 0.38-1.18 | 0.17 | 53 |
| CD8+ cells /mm^2^ transition zone | 0.73 | 0.42-1.28 | 0.27 | 52 |
| CD8+ cells/mm^2^ periphery | 1.25 | 0.66-2.37 | 0.50 | 43 |
| FOXP3+ cells/mm^2^ core | 0.97 | 0.56-1.68 | 0.92 | 53 |
| FOXP3+ cells/mm^2^ transition zone | 1.01 | 0.58-1.75 | 0.98 | 52 |
| FOXP3+ cells/mm^2^ periphery | 0.79 | 0.43-1.47 | 0.46 | 43 |
| IBA1+ cells/mm^2^ core | 1.11 | 0.64-1.91 | 0.71 | 53 |
| IBA1+ cells/mm^2^ transition zone | 0.90 | 0.52-1.56 | 0.70 | 52 |
| IBA1+ cells/mm^2^ periphery | 0.99 | 0.54-1.84 | 0.98 | 43 |
| **Tumor cell proximity** |  |  |  |  |
| Tumor cells/mm^2^ with 1-4 CD8+ cells in proximity, core | 0.93 | 0.54-1.61 | 0.80 | 53 |
| Tumor cells/mm^2^ with 1-4 CD8+ cells in proximity, transition zone | 0.94 | 0.54-1.64 | 0.83 | 50 |
| Tumor cells/mm^2^ with 1-4 CD8+ cells in proximity, periphery | 1.51 | 0.82-2.82 | 0.19 | 43 |
| Tumor cells/mm^2^ with 1-4 FOXP3+ cells in proximity, core | 1.01 | 0.59-1.75 | 0.96 | 53 |
| Tumor cells/mm^2^ with 1-4 FOXP3+ cells in proximity, transition zone | 1.02 | 0.58-1.79 | 0.94 | 52 |
| Tumor cells/mm^2^ with 1-4 FOXP3+ cells in proximity, periphery | 1.13 | 0.65-1.95 | 0.66 | 54 |
| Tumor cells/mm^2^ with 1-4 IBA1+ cells in proximity, core | 0.92 | 0.53-1.60 | 0.77 | 53 |
| Tumor cells/mm^2^ with 1-4 IBA1+ cells in proximity, transition zone | 0.70 | 0.39-1.24 | 0.22 | 52 |
| Tumor cells/mm^2^ with 1-4 IBA1+ cells in proximity, periphery | 1.64 | 0.88-3.04 | 0.12 | 43 |
| Tumor cells/mm^2^ with 5-9 IBA1+ cells in proximity, core | 1.03 | 0.60-1.78 | 0.92 | 53 |
| Tumor cells/mm^2^ with 5-9 IBA1+ cells in proximity, transition zone | 0.73 | 0.42-1.28 | 0.28 | 52 |
| Tumor cells/mm^2^ with 5-9 IBA1+ cells in proximity, periphery | 1.16 | 0.63-2.14 | 0.63 | 42 |
| Tumor cells/mm^2^ with over 10 IBA1+ cells in proximity, core | 0.94 | 0.54-1.63 | 0.82 | 53 |
| Tumor cells/mm^2^ with over 10 IBA1+ cells in proximity, transition zone | 0.57 | 0.31-1.07 | 0.08 | 54 |
| Tumor cells/mm^2^ with over 10 IBA1+ cells in proximity, periphery | All recorded measurements are 0. |  |  |  |

Tumor cells/mm^2^ with over 10 IBA1+ cells in proximity, transition zone: A substantial number of cases have a value of 0. We have categorized them into two groups: 0 and >0. The group with a score of 0 comprises 40 cases, while the group with a higher score includes 13 cases.

Tumor cells/mm^2^ with 1-4 FOXP3+ cells in proximity, periphery: A substantial number of cases have a value of 0. We have categorized them into two groups: 0 and >0. The group with a score of 0 comprises 26 cases, while the group with a higher score includes 28 cases.
